# The Dyadic Universe Model: a process theory of centralization, compression, and autonomy in romantic relationships

**DOI:** 10.3389/fpsyg.2026.1935191

**Published:** 2026-10-19

**Authors:** Yuyang Yan, Jing Li

**Affiliations:** School of Education, Guangzhou University, Guangzhou, China

**Keywords:** autonomy restoration, dispositional relational fluidity, dyadic centralization, existential expansion need, explanatory gap in relationship theories, identity fusion, lifecycle phases (recognition, centralization, saturation, autonomy restoration), partner-mediated identity restructuring

## Abstract

Contemporary relationship science provides rich accounts of attachment, intimacy, and self-expansion, yet remains comparatively underspecified regarding a class of high-intensity romantic relationships in which partners progressively become each other's primary source of identity and meaning. We introduce the Dyadic Universe Model (DUM), a process-theoretic framework developed to address this explanatory gap, since existing theories such as attachment, self-expansion, differentiation of self, and the suffocation model explain important aspects of closeness, growth, or strain but do not fully capture the distinctive pattern in which expansion later turns into perceived constraint. This framework is formalized using three core variables: dyadic centralization (C), external-world investment (E), and dispositional pressure (ψ). Dyadic centralization captures the extent to which the relationship displaces external domains of identity and engagement; external world investment reflects ongoing participation in those domains; and dispositional pressure represents individual differences in the drive for novelty, growth, and identity diversification. The model proposes a recursive dynamic in which increasing centralization is associated with reduced external engagement, limiting access to novel inputs and contributing to the accumulation of subjective compression. For individuals higher in dispositional pressure, this compression is predicted to intensify over time, eventually activating autonomy-restoration processes that reduce centralization or destabilize the relationship. From this minimal structure, the model derives a characteristic four-phase lifecycle, which are recognition, centralization, saturation, and autonomy restoration, linking the emergence of exceptional relational intensity to its potential transformation into psychological constraint. The DUM integrates and extends existing theories of self-expansion, differentiation of self, and relational suffocation by specifying the temporal dynamics through which immersive relationships evolve. It advances a set of falsifiable propositions concerning lifecycle trajectories, individual differences, and dyadic asymmetries, and outlines a coordinated empirical research program across longitudinal, dyadic, and experience-sampling designs. While elements of these dynamics are suggested across existing empirical findings, the model formalizes them into a unified process-level framework and generates explicit predictions requiring systematic empirical testing. By articulating the interplay between centralization, external engagement, and dispositional pressure, the DUM provides a parsimonious account of how the same processes that generate profound intimacy may also create conditions for instability.

## 1 Introduction

Romantic relationships organize substantial portions of adult identity, affect regulation, and meaning making (Berscheid, 1999; Finkel et al., 2017). Decades of research have produced sophisticated accounts of attachment processes (Mikulincer and Shaver, 2016), interdependence and commitment (Rusbult et al., 2012), perceived partner responsiveness (Reis, 2014), self-expansion (Aron et al., 2013), and the long-term trajectories of passionate love (Acevedo and Aron, 2009). Recent work has also highlighted how trauma and dysfunctional attachment dynamics shape apparently normal relationship styles (Kaşikçi and Öztürk, 2026), and how doctrines of self-actualization can extend beyond psychotherapies to relational contexts (Öztürk, 2025). Yet converging observations across clinical accounts, qualitative reports, and selected empirical findings suggest a pattern that remains incompletely theorized: some dyads do not merely become close—they become, for a period, each other's primary reality.

Participants in such relationships report unusually rapid psychological recognition, heightened relational priority over other life domains, and a paradoxical coexistence of profound meaning with mounting destabilization. Neuroscience studies demonstrate that the dorsomedial prefrontal cortex can evaluate potential romantic partners within milliseconds, predicting attraction outcomes, while neurochemical cascades involving dopamine and norepinephrine explain the intense feelings often described as “love at first sight” (Cooper et al., 2012; Ceruto, 2026). Psychological research further highlights neural synchrony and language style matching as predictors of rapid relational bonding, underscoring how accelerated recognition can coexist with destabilizing intensity (Ture, 2025). This duality resonates with perspectives on betrayal trauma, which show how relational bonds can simultaneously provide profound meaning and generate psychological distress (Freyd et al., 2005). Popular discourse captures these dynamics with terms such as “soulmate” or “once-in-a-lifetime,” yet academic psychology has been reluctant to formalize such labels. While the soulmate concept is culturally pervasive, empirical research does not support predestined matches, instead attributing these experiences to neurochemical bonding and cognitive biases such as the halo effect (Bennett, 2025; NeuroLaunch Editorial Team, 2024). This reluctance is warranted; however, the underlying empirical phenomenon remains in need of systematic explanation.

Several strands of empirical research indirectly point to this pattern. Research on enmeshment and relational overdependence demonstrates that excessive involvement within close relationships often blurs boundaries, thereby constraining autonomy and broader identity functioning (Bacon and Conway, 2023; Martin, 2025; Simply Psychology Editorial, 2026). While work on long-term love suggests that intense bonds can persist alongside diminishing novelty, with novelty-seeking shown to be critical for sustaining satisfaction (González-Cutre et al., 2020). Emerging perspectives even situate relational intensity within broader socio-cultural and trauma frameworks, such as necriphysiphilia as a psychopathology of the Anthropocene epoch (Öztürk, 2024). Additionally, research on infidelity indicates that autonomy-seeking behaviors may emerge even in relationships that individuals describe as meaningful, often as a way to preserve independence or cope with unmet needs (Alpysbekova, 2025). These findings are typically treated separately but collectively suggest a dynamic in which intense relational centrality may coexist with, and eventually contribute to, experiences of constraint.

Existing theoretical frameworks do not fully integrate important components of this phenomenon but do not fully integrate them into a unified account of how relationships transition from expansion to perceived constraint over time. Attachment theory explains patterns of dependency and separation distress but does not specify why psychologically secure individuals may enter relationships that reorganize identity around a single partner (Bowlby, 1982). Self-expansion theory explains the intrinsic rewards of incorporating another into the self, but leaves underspecified the transition from expansion to perceived constriction (Aron and Aron, 1986). Differentiation-of-self frameworks anticipate the costs of fusion but treat these primarily as trait-based capacities rather than dynamically evolving processes (Bowen, 1978). The suffocation model of marriage identifies rising relational demands but locates their origin primarily in cultural expectations rather than in endogenous dyadic dynamics (Finkel et al., 2015).

We propose that these observations reflect a distinct relational regime characterized by recursive psychological processes operating within the dyad. In this regime, increasing relational centrality progressively attenuates engagement with external domains of identity and meaning. This attenuation, particularly for individuals with strong dispositional drives toward novelty and expansion, generates increasing psychological pressure that may ultimately destabilize the relationship. Intensity and fragility, on this account, are not opposing tendencies but co-products of the same underlying dynamics. This perspective resonates with Öztürk's (2024) analysis of necriphysiphilia, which situates relational intensity within broader socio-cultural and trauma frameworks, showing how individual experiences of compression can scale into collective patterns of constraint.

The Dyadic Universe Model (DUM) formalizes this process-theoretic account. The model is developed in a staged manner to balance conceptual accessibility with theoretical precision. We begin with a minimal core representation that captures the essential dynamics of the system, then extend this framework to a richer set of constructs and a formal dynamical structure. From this foundation, we derive a set of falsifiable propositions that specify the conditions under which these dynamics should emerge. To maintain accessibility, we first introduce the model conceptually, emphasizing its minimal structure and intuitive dynamics. A formal definition of the variables and their interactions is provided in Section 6, where the full generative system is specified.

Having established that high-intensity dyads are documented across clinical case reports, qualitative interviews, and everyday relational accounts, we now turn to the major theoretical traditions in relationship science to clarify where existing frameworks succeed, where they fall short, and why a process-level account, such as the Dyadic Universe Model, is needed.

## 2 Clinical and qualitative evidence for high-intensity dyads

The Dyadic Universe Model is motivated by converging evidence across clinical case reports, qualitative interview studies, and everyday relational accounts. These sources consistently document the sequence of rapid recognition, relational centralization, saturation, and autonomy-restoration that the model formalizes.

### 2.1 Clinical accounts

Psychotherapy literature has long described couples whose relationships become unusually immersive, often producing both profound meaning and destabilization. Case studies of betrayal trauma highlight how bonds can simultaneously provide security and generate distress when relational immersion constrains broader identity functioning (Freyd et al., 2005). Clinical research on enmeshment and destructive overdependence demonstrates that relational immersion often displaces external domains of identity, producing both closeness and constraint in functioning (Bornstein and Thomson, 2024).

Recent clinical research further substantiates these dynamics. Papa et al. (2026) identify Pathological Affective Dependence (PAD) and Fear of Intimacy (FoI) as relational conditions in which bonds become immersive yet destabilizing. PAD reflects the drive to preserve closeness at all costs, while FoI reflects defensive autonomy restoration. Both conditions illustrate the recursive dynamics of centralization and compression formalized in the DUM. Similarly, (Incekara and Ulaş 2026) conceptualize romantic relationship addiction as a maladaptive dependence with addiction-like features, showing how relational immersion can become compulsive and destabilizing—a clinical analog of the DUM's saturation phase. Complementing these findings, Molina et al. (2026) provide a systematic review of co-dependency, highlighting disrupted identity and impaired functioning when relational immersion displaces external domains. These outcomes exemplify the DUM's account of external attenuation and subjective compression.

Taken together, these clinical accounts demonstrate that the target phenomenon is not merely interpretive synthesis but is empirically documented across multiple strands of psychotherapy and clinical psychology. They provide concrete evidence that immersive dyads follow a recognizable sequence of recognition, centralization, saturation, and autonomy-restoration, thereby validating the DUM's process-theoretic framework.

### 2.2 Qualitative reports

Interview-based studies of romantic narratives reveal that individuals often describe “soulmate” or “once-in-a-lifetime” relationships as characterized by rapid psychological recognition and heightened relational priority (Bennett, 2025; NeuroLaunch Editorial Team, 2024). Ethnographic and qualitative analyses of couple identity show how partners progressively adopt collective language (“we-talk”) and reorganize daily life around the relationship, sometimes at the expense of external pursuits (Agnew et al., 1998; Selcuk et al., 2017).

### 2.3 Everyday reports

Survey and diary-based studies provide evidence that intense relational bonds can coexist with diminishing novelty and eventual autonomy-seeking. Longitudinal research on passionate love shows that while intensity may remain high, novelty declines over time, producing restlessness or constraint (Acevedo and Aron, 2009). Everyday accounts of infidelity further illustrate autonomy-restoration behaviors emerging even in relationships described as deeply meaningful (Alpysbekova, 2025).

Taken together, these clinical, qualitative, and everyday sources establish that the target phenomenon is not merely speculative. Rather, it reflects a recurring relational pattern documented across multiple literatures. The Dyadic Universe Model builds on this foundation by formalizing the recursive processes through which relational centrality, external attenuation, and dispositional pressure interact to generate both intensity and fragility.

## 3 Conceptual foundations and the explanatory gap

Contemporary relationship science offers multiple well-developed frameworks for understanding how romantic relationships form, deepen, and sometimes deteriorate. Each of these frameworks explains important parts of relational experience, but none fully accounts for a particular pattern observed across clinical, qualitative, and everyday reports: relationships in which partners become each other's primary reality, followed by a later sense of psychological constriction and instability.

This section reviews the major theoretical traditions relevant to this phenomenon and identifies a specific explanatory gap. The goal is not to replace existing theories, but to clarify where they stop and why an additional process-level account is needed.

### 3.1 Attachment and its limits for high-intensity systems

Attachment theory remains one of the most influential frameworks in relationship science. It explains how individuals regulate closeness and distance, particularly under conditions of threat or insecurity (Hazan and Shaver, 1987; Mikulincer and Shaver, 2016). Anxiously attached individuals tend to seek closeness intensely, while avoidant individuals tend to distance themselves. The theory has been widely supported and has contributed to measurement tools, neuroscience findings, and therapeutic approaches.

However, attachment theory is primarily a theory of dependency regulation, not of relationship structure. It explains how people respond to closeness, but not why two individuals, especially psychologically secure individuals, might voluntarily organize their lives so that one relationship dominates most aspects of identity and meaning.

In many high-intensity relationships, both partners may be relatively secure yet still become deeply immersed in each other. This suggests that the phenomenon is not simply about insecurity or hyperactivation. Rather, it involves a shift in how the relationship is positioned within a person's overall life system. In simple terms, attachment theory explains emotional reactions within a relationship, but not why the relationship itself becomes everything.

### 3.2 Self-expansion and the unspecified transition

Self-expansion theory proposes that people are motivated to grow by incorporating others' perspectives, skills, and identities into their own (Aron and Aron, 1986; Aron et al., 2013). Romantic relationships are especially powerful in providing this sense of growth, which helps explain the excitement and meaning often felt in early stages. This framework is strongly supported by evidence showing that couples who engage in shared, novel activities report higher relationship quality (Aron et al., 2000; Muise et al., 2019). However, a key puzzle remains: If relationships are a source of growth, why do some of them later feel restrictive rather than expansive? While later work has distinguished between positive self-change (expansion) and negative self-change (contraction) (Mattingly and Lewandowski, 2014), it does not fully explain how the same relationship shifts from one to the other over time.

A simple way to understand this gap is through everyday experience. A couple may initially feel that they are discovering new aspects of themselves together. Over time, however, if most of their time, attention, and identity becomes centered only on each other, they may begin to feel stuck despite still caring deeply for one another. In summary, self-expansion theory explains how relationships help people grow, but not why that same process sometimes turns into a feeling of being limited.

### 3.3 Differentiation of self and couple identity

The concept of differentiation of self (Bowen, 1978) focuses on a person's ability to maintain a clear sense of identity while staying emotionally connected to others. Individuals with low differentiation may become overly fused with their partner's emotions and perspectives, while highly differentiated individuals can balance closeness with independence. Related research on couple identity shows that partners often begin to think and speak in more collective terms (“we” instead of “I”), and that this can be beneficial for relationship stability (Agnew et al., 1998; Slatcher and Schoebi, 2017). These frameworks are highly valuable. However, they largely treat fusion as either: (1) a trait-like tendency (in differentiation), or (2) a generally adaptive outcome (in couple identity research). They do not strongly address the situation in which couple identity becomes so dominant that other aspects of life, such as career, friendships, individual goals, are gradually reduced. In essence, these theories tell us what it means to stay “balanced,” but they do not fully explain how or why some relationships move beyond balance into complete centrality. At a deeper level, differentiation of self is not only about managing closeness with a partner but about sustaining a coherent inner identity that remains stable even under intense emotional immersion. Bowen (1978) emphasized that the self is a developmental achievement—an ability to think, feel, and act from one's own principles while staying connected to others. When differentiation is weak, the self can become absorbed into the partner's emotional field, leading to fusion that erodes individuality. Conversely, a well-differentiated self allows individuals to experience intimacy without losing their personal boundaries, goals, or values. This deeper view highlights that the “self” is not a static trait but a living system that must continually negotiate between autonomy and connection.

### 3.4 Identity fusion as a related but distinct construct

The concept of identity fusion (Swann et al., 2009, 2012) describes a powerful sense of oneness with a group, often leading to strong loyalty and even self-sacrifice. At first glance, this may seem similar to what occurs in highly intense romantic relationships. However, there are important differences:

- Identity fusion usually involves a group, while this paper focuses on a single partner;
- It is driven by shared group identity, rather than ongoing mutual interaction; and
- Its outcomes are typically pro-group actions, not changes in personal life structure.

Thus, while the two ideas share the theme of blurred boundaries between self and other, they operate in different contexts and through different mechanisms. All in all, identity fusion helps explain intense group loyalty but does not directly explain why two people reorganize their entire lives around each other. At the same time, dissociation literature offers a useful parallel. Dissociation refers to disruptions in the normal integration of memory, identity, and consciousness (Putnam, 1997; Dell and O'Neil, 2009). In highly immersive relational contexts, the boundaries of self may blur in ways that resemble dissociative processes. This is where aspects of identity are absorbed into the partner or the dyad, reducing access to independent self-functions. While identity fusion emphasizes unity and loyalty, dissociation highlights fragmentation and altered integration. Considering these together suggests that extreme relational immersion may involve both fusion (a sense of oneness) and dissociative tendencies (a weakening of autonomous self-integration). This connection underscores why the Dyadic Universe Model must account not only for closeness but also for the potential erosion of self-boundaries over time.

### 3.5 The suffocation model of marriage

The suffocation model (Finkel et al., 2014a,b; Finkel, 2017) argues that modern relationships are expected to fulfill a wide range of needs—from basic companionship to self-actualization. Because expectations are so high, many relationships struggle to meet them. This model comes closest to the phenomenon of interest. It highlights how relationships can become overwhelming under increasing demands. However, it primarily explains this pressure as coming from cultural and societal changes. For example, modern expectations about love and fulfillment. The present paper takes a different perspective. It proposes that, even without cultural pressure, some relationships develop internal dynamics that naturally lead to high intensity and eventual strain. Summing up, the suffocation model explains why relationships are under pressure, but less so how that pressure emerges from within the relationship itself.

Beyond cultural expectations, self-actualization perspectives highlight how relationships themselves can become vehicles for existential growth. (Öztürk 2025) argues that doctrines of self-actualization extend beyond psychotherapies into relational contexts, suggesting that partners often seek not only companionship but also personal transformation through the bond. This pursuit of growth can initially enhance meaning and vitality, yet when external domains of identity are reduced, the same drive may intensify feelings of constraint. In simple terms, the very search for fulfillment within the relationship can create pressures that mirror, and sometimes exceed, those described by the suffocation model. Integrating self-actualization literature therefore clarifies how internal dynamics of growth and compression interact with cultural demands, producing both the promise and strain of modern high-intensity relationships.

### 3.6 Passionate love, novelty, and decline

Research on passionate love shows that intense emotional states often decline over time (Acevedo and Aron, 2009). At the same time, some couples maintain strong emotional bonds without the initial intensity fading completely. This body of work highlights an important constraint: “Any theory of relationships must explain what declines, what remains stable, and what changes form over time.” However, this literature does not fully explain the specific pattern in which intensity remains high; but a feeling of restriction or restlessness increases. Simply put, it shows that love changes over time, but not exactly why some intense relationships become psychologically constraining.

### 3.7 Dynamical systems approaches

Dynamical systems approaches (Gottman et al., 2002; Vallacher et al., 2005) view relationships as evolving systems, rather than static traits. These models have successfully explained patterns such as stability, instability, and feedback loops in couple interactions. The current model builds on this tradition but differs in two ways. First, it focuses on long-term identity-level processes, rather than moment-to-moment interactions. Second, it introduces mechanisms to explain how major shifts (such as breakup) emerge over time, rather than being assumed. In brief, dynamical models show that relationships evolve over time—but do not yet specify the particular processes that lead to the pattern of intense centralization followed by compression.

### 3.8 Synthesis and the explanatory gap

Taken together, existing theories provide a rich understanding of relationships, but each addresses only part of the phenomenon.

- Attachment explains emotional regulation within relationships.
- Self-expansion explains how relationships promote growth.
- Differentiation explains the balance between closeness and individuality.
- Identity fusion explains strong self–other overlap in groups.
- The suffocation model explains cultural pressure on relationships.
- Dynamical systems models explain how relationships evolve over time.

Yet a specific set of observations remains only partially explained across existing frameworks:

1. Rapid centralization: Why do some relationships quickly become central to a person's entire sense of identity, even when both partners are psychologically secure?
2. Expansion turning into compression: Why does a relationship that initially promotes growth later feel restrictive or limiting, without a clear external cause?
3. Autonomy-seeking after peak closeness: Why do behaviors such as distancing, novelty-seeking, or even rupture often emerge after the relationship has reached its deepest closeness, rather than during weaker phases?
4. Individual differences: Why do these patterns occur more strongly in some individuals than in others?

These questions all point to a common missing element: a process-level account of how relationships reorganize a person's broader life system over time.

The Dyadic Universe Model is developed to address this gap. The term “Dyadic Universe” was coined to emphasize how certain relationships evolve into a self-contained world of two. The word “dyadic” highlights that the phenomenon is specific to a pair, while “universe” conveys the encompassing scope of identity, meaning, and emotional regulation that the relationship comes to dominate. This name reflects the model's concern with how such immersive relational worlds generate both profound intimacy and eventual psychological compression. It proposes that the same processes that generate deep connection can, under certain conditions, gradually reduce engagement with the wider world, leading to a sense of psychological compression and eventual instability. The model begins with a minimal core structure, from which more detailed constructs and predictions are derived, ensuring both conceptual clarity and empirical testability. Addressing this gap requires a framework that specifies how relational centrality, external engagement, and individual expansion drives interact dynamically over time. To that end, we now introduce a minimal generative model that captures these interactions in a parsimonious form.

## 4 Minimal core model of the dyadic universe model

To establish conceptual clarity and accessibility, we begin with a minimal core model built on three variables. The remaining constructs, definitions, and propositions are retained as extensions and elaborations derived from this core.

### 4.1 Definition

A dyadic universe is defined as a relational regime in which two partners become each other's primary source of identity, meaning, and emotional regulation, to the extent that engagement with external domains (e.g., social networks, work, personal pursuits) is substantially reduced.

This regime is not universal to all relationships but represents a distinct structural configuration characterized by high internal immersion and reduced external integration.

### 4.2 The three core elements

The minimal core model is built on three variables:

- **Dyadic centralization (C)**

The extent to which the relationship displaces external domains of identity, attention, and engagement.

- **External-world investment (E)**

The degree of ongoing participation in social, occupational, and identity-relevant domains outside the relationship.

- **Dispositional pressure (ψ)**

An individual-level tendency toward novelty, growth, and diversification of identity, reflecting drives described in self-expansion theory (Aron et al., 2013). Importantly, dispositional pressure is not reducible to general personality traits such as openness to experience; rather, it refers specifically to the drive toward relationally relevant novelty and identity diversification.

### 4.3 Core dynamics

The interaction of these variables produces a recursive system:

1. **Centralization increases (C** **↑)** as partners invest more in the relationship
2. **External investment decreases (E** **↓)** due to limited time and attention
3. **Reduced external engagement limits access to novelty and growth**
4. **Subjective compression develops**, especially for individuals high in ψ
5. **Pressure activates corrective responses**, reducing centralization or destabilizing the relationship

This produces the feedback loop:

**C** **↑** **→** **E** **↓** **→** **reduced novelty** **→** **pressure (ψ)** **↑** **→** **C adjusted**

We refer to this as the central recursive principle of the Dyadic Universe Model: increasing centralization reduces external engagement, thereby generating pressure that ultimately acts back upon centralization itself.

This minimal structure explains how relationships can become intensely meaningful while also generating the conditions for later strain. For formalization purposes, subjective compression is represented as an accumulated phenomenological state variable K(t), capturing the dynamic buildup of perceived restriction.

Although the verbal description of the Dyadic Universe Model (DUM) may resemble a sequential mediation chain (centralization → reduced external investment → compression → autonomy-restoration), the model is formally specified as a nonlinear feedback system. Autonomy-restoration behaviors are not merely outcomes but recursive inputs: when enacted, they alter subsequent levels of dyadic centralization and external-world investment. For example, successful autonomy-restoration reduces centralization intensity and re-opens external investment opportunities, while unsuccessful attempts can heighten dispositional pressure (ψ), reinforcing compression. Thus, the system operates as a recursive dynamical process rather than a unidirectional chain, with feedback loops generating oscillatory or convergent trajectories over time. This distinction aligns with broader systems theory perspectives that emphasize recursive causality and dynamic adjustment cycles in relational and organizational contexts (Bertalanffy, 1968; Coleman, 1990). It is further reflected in the stability regimes derived in Appendix A, where eigenvalue analyses demonstrate how autonomy-restoration feeds back into centralization and external engagement, producing either damped oscillations or overdamped convergence depending on ψ.

Figure 1 presents the conceptual framework of the Dyadic Universe Model (DUM), which operates as a nonlinear feedback system. The diagram visualizes how centralization (C) increases dyadic investment but simultaneously reduces external investment (E). This reduction limits novelty and growth, producing subjective compression (K), and elevating dispositional pressure (ψ). Heightened pressure activates autonomy restoration behaviors (AR), which feed back into the system by modifying subsequent levels of centralization and external investment. Successful autonomy restoration decreases C and reopens E, while unsuccessful attempts intensify ψ, reinforcing compression and centralization. These recursive feedback loops generate oscillatory or convergent trajectories over time, consistent with the stability regimes derived in Appendix A. The nonlinear feedback structure ensures that the DUM captures dynamic adjustment cycles, where outputs at one stage become inputs at the next, reflecting the self-regulating nature of dyadic relationships. This specification aligns with systems theory perspectives emphasizing recursive causality and dynamic equilibrium in complex social systems (Bertalanffy, 1968; Coleman, 1990).

**Figure 1:**
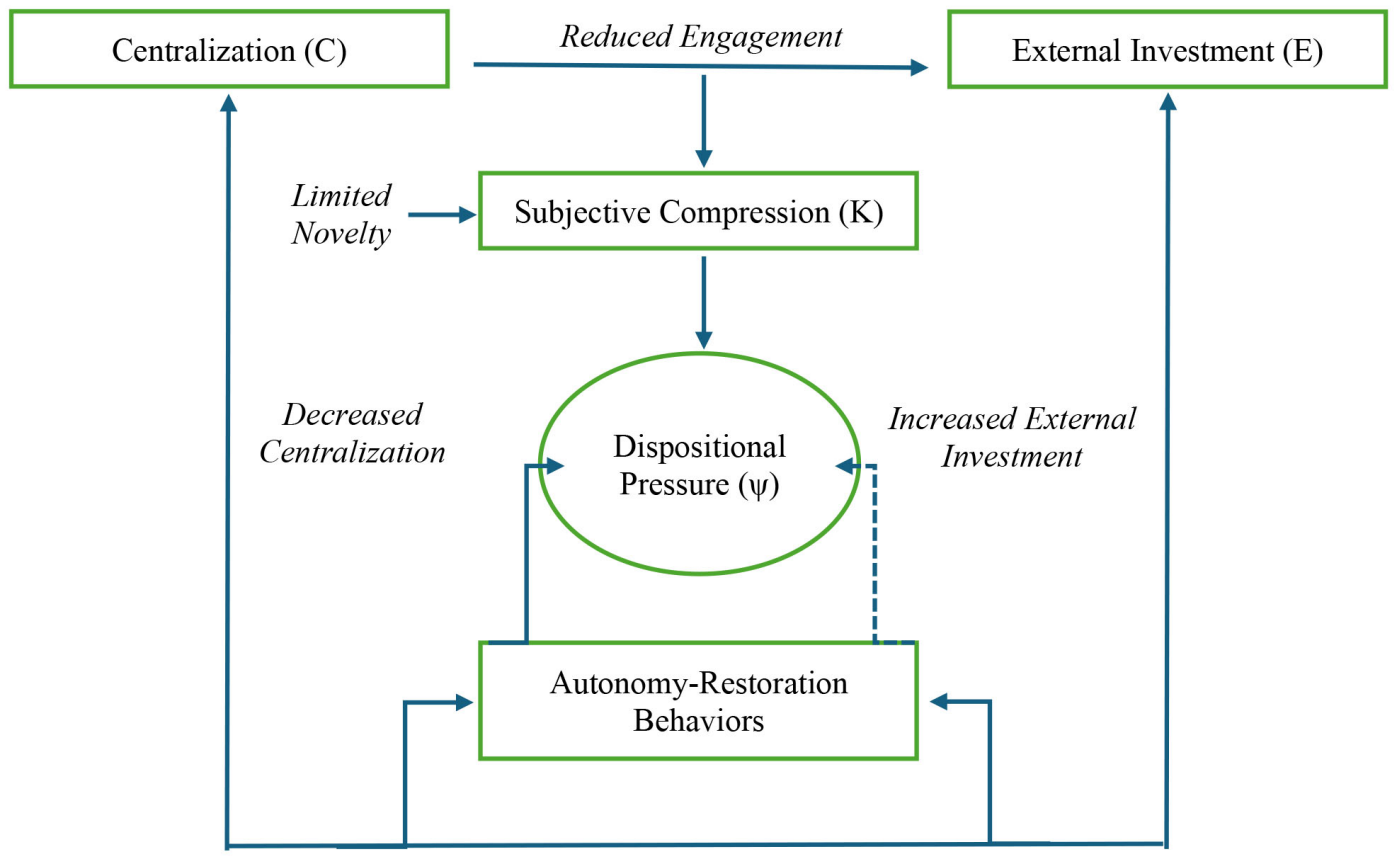
Conceptual framework of the Dyadic Universe Model (DUM) characterized by a recursive feedback structure in a nonlinear feedback system.

### 4.4 Expanded constructs (derived from the core model)

The five constructs of the full DUM are retained as extensions and decompositions of C, E, and ψ:

1. **Relational salience**. Represents the cognitive and attentional dominance of the relationship; functions as a proximal expression of increasing centralization (C).
2. **Partner-mediated identity restructuring**. Describes how partners actively reshape each other's self-concept; serves as a mechanism through which **centralization (C)** develops and deepens.
3. **Dyadic centralization (C)**. Retained as a core construct, structurally capturing displacement of external domains.
4. **Dispositional relational fluidity**. Represents the tendency toward novelty-seeking in relational contexts; forms a component of dispositional pressure (ψ).
5. **Existential expansion need**. Reflects the importance of ongoing growth and becoming; contributes to dispositional pressure (ψ).

Together, these constructs provide measurement-ready components of the minimal variables while preserving conceptual parsimony.

### 4.5 Discriminant validity positioning

Each construct is conceptually distinct from existing frameworks:

- **Centralization (C)** differs from:

° Attachment (focus on emotional regulation; Mikulincer and Shaver, 2016).
° Couple identity (linguistic or symbolic unity; Slatcher and Schoebi, 2017).
° Identity fusion (group-based merging; Swann et al., 2009).

- **External investment (E)** differs from:

° Satisfaction or commitment (Rusbult et al., 2012), as it captures structural engagement beyond the relationship.

- **Dispositional pressure (ψ)** differs from:

° Openness or curiosity, by focusing specifically on relationally relevant expansion needs.

These distinctions ensure that the model captures a structural-process mechanism, rather than re-labeling existing traits or outcomes. Table 1 specifies the discriminant validity claims that an empirical program must establish.

**Table 1:** Discriminant validity claims for DUM constructs.

DUM construct	Nearest existing construct(s)	Discriminant claim
Relational salience	Attachment anxiety; passionate love	Cognitive/existential occupation independent of threat activation or hedonic affect
Partner-mediated identity restructuring	Inclusion-of-other-in-self; perceived partner responsiveness	Active self-concept restructuring; requires recipient susceptibility, not only partner behavior
Dyadic centralization	We-talk; couple identity; Swann's identity fusion	Concerns relative weighting of dyad vs. external domains; dyad-specific (not collective); structural rather than motivational
Dispositional relational fluidity	Openness; sociosexual orientation; Behavioral Activation System (BAS)	Domain-specific drive toward novelty and external-world engagement, distinct from sexual behavior *per se*
Existential expansion need	Need for growth; trait curiosity	Specifically future-oriented horizon-widening; distinct from autonomy or epistemic seeking

### 4.6 Core propositions (derived from the minimal model)

From the interaction of C, E, and ψ, the following propositions are derived:

**P1. Centralization–external trade-off**. Increases in dyadic centralization will correspond to decreases in external-world investment.

**P2. Nonlinear intensification**. Entry into high-centralization relationships will show rapid escalation rather than gradual increase.

**P3. External-domain attenuation**. High centralization predicts reduced diversity in social, occupational, and identity domains.

**P4. Expansion-to-compression transition**. Initial self-expansion through the relationship will shift to perceived restriction under sustained external attenuation.

**P5. Inverted-U effect of centralization**. Relationship vitality increases with centralization up to a point, then declines as compression emerges.

**P6. Pressure sensitivity**. Individuals higher in dispositional pressure (ψ) will experience greater distress at equivalent levels of centralization.

**P7. Autonomy-restoration tendency**. Corrective behaviors (reinvestment, distancing, or rupture) are more likely after peak centralization.

**P8. Outcome moderation**. Whether autonomy-restoration is constructive or destabilizing depends on relational norms regarding external engagement.

Additional propositions (including dyadic asymmetry, rupture initiation, and stochastic outcomes) are retained in the extended formal model.

### 4.7 Emergent lifecycle

The interaction of C, E, and ψ generates a characteristic four-phase trajectory:

1. **Recognition** – rapid relational salience and identity activation.
2. **Centralization** – increasing dominance of the relationship (C ↑, E ↓).
3. **Saturation** – reduced novelty and increasing compression
4. **Autonomy restoration** – rebalancing or destabilization driven by ψ.

### 4.8 Summary of the integrated model

The minimal core model, defined by C, E, and ψ, provides a parsimonious explanation for:

- Rapid emergence of intense relational immersion.
- Transition from growth to perceived restriction.
- Individual differences in relational stability.
- The coexistence of deep intimacy and structural fragility.

The expanded constructs, discriminant structure, and propositions are retained as measurement, validation, and empirical extensions, all anchored in this minimal generative system.

C ↑ → E ↓ → K ↑ → Autonomy-restoration → C adjusted.

## 5 Lifecycle dynamics

The dynamics described in the minimal core model (Section 3) generate a predictable temporal trajectory. As dyadic centralization (C) increases and external-world investment (E) declines, the system progressively alters the individual's experiential landscape. For individuals with moderate to high dispositional pressure (ψ), this process produces a sequence of psychologically distinct phases.

These phases are not independent stages imposed externally; rather, they emerge directly from the recursive structure of the system. The same mechanisms that generate initial relational intensity gradually produce the conditions for later compression and autonomy-restoration.

For clarity and empirical tractability, the model specifies four phases: recognition, centralization, saturation, and autonomy restoration.

### 5.1 Phase 1: recognition and acceleration

The relationship feels unusually meaningful and significant very quickly.

The relationship begins with subjectively rapid mutual identification. Individuals report early interactions as unusually consequential, often accompanied by a sense of immediate clarity or “fit” with the partner. From a process standpoint, this phase reflects a rapid increase in relational salience and the early activation of partner-mediated identity restructuring.

Consistent with prior findings, perceived similarity, reciprocal self-disclosure, and contingent responsiveness contribute to this effect (Aron et al., 1997; Sprecher, 2014). What distinguishes this phase within the present model is not the presence of these mechanisms, but their rate of escalation, which is predicted to be steeper for individuals with higher relational salience capacity and responsiveness.

At this stage, external-world investment (E) remains relatively intact, and the relationship is experienced primarily as expansive rather than constraining.

### 5.2 Phase 2: centralization

The relationship becomes the main center of life, typically in a positive and meaningful way. Dyadic centralization (C) increases as the relationship assumes greater priority in identity, attention, and daily life organization. External domains, such as friendships, career pursuits, and independent activities, begin to lose relative weighting, leading to a corresponding decline in E.

This phase is typically experienced as highly rewarding. The partner becomes a primary source of meaning-making, and the mechanisms described by self-expansion theory are at or near peak intensity (Aron et al., 2013). The relationship facilitates growth by providing access to new perspectives, identities, and experiences mediated through the partner.

However, the model identifies a critical structural feature:

The same process that drives expansion also begins to attenuate the external domains from which future expansion would otherwise arise.

In effect, the system becomes increasingly self-referential. The relationship grows by drawing from external sources, but in doing so gradually reduces those sources' future availability. This recursive dynamic constitutes the engine of the dyadic-universe regime.

### 5.3 Phase 3: saturation

The relationship still matters deeply, but life begins to feel narrower or less dynamic.

As centralization remains high and external investment continues to decline, the system enters a saturation phase. Partner-mediated identity restructuring persists but yields diminishing returns due to reduced access to novel external inputs.

This transition is predicted to correspond to a shift from self-expansion to self-contraction (Mattingly and Lewandowski, 2014). The individual's experiential horizon narrows, particularly for those high in dispositional pressure (ψ), whose motivational structure places greater emphasis on novelty, growth, and diversification.

The subjective experience of compression emerges during this phase. It often presents initially as diffuse restlessness, reduced excitement, or a sense of stagnation, rather than as explicitly relationship-focused dissatisfaction. Importantly, the model predicts that: “Compression and relationship satisfaction can co-occur.”

Individuals may simultaneously report deep emotional connection and growing psychological constraint. This dissociation reflects the model's claim that relational quality and systemic structure are not identical constructs.

### 5.4 Phase 4: autonomy-restoration and trajectory bifurcation

One or both partners begin trying to create space or reintroduce independence.

As subjective compression accumulates, autonomy-restoration processes are activated (P7). These processes are hypothesized to represent attempts to rebalance the system by increasing external-world engagement (E) and reducing excessive centralization (C).

Autonomy-restoration behaviors vary in form and consequence. Constructive variants include:

- Reinvestment in external social or occupational domains
- Engagement in identity-diversifying activities
- Explicit renegotiation of boundaries within the relationship

In contrast, destabilizing variants include:

- Emotional distancing
- Externalized validation seeking
- Extra-dyadic relational involvement

Which pathway emerges depends in part on relational norms governing the legitimacy of external engagement (P8), as well as on the partner's response to autonomy-restoration attempts.

This phase therefore constitutes a bifurcation point:

In one trajectory, the dyad successfully recalibrates centralization, allowing for a sustainable equilibrium characterized by moderated C and restored E.

In the alternative trajectory, autonomy-restoration efforts are misaligned, resisted, or externalized in destabilizing ways, increasing the likelihood of rupture or dissolution.

### 5.5 Integrative note on lifecycle dynamics (new closing paragraph)

Across all four phases, a single principle governs the system: “The processes that generate exceptional closeness also progressively limit the conditions required to sustain that closeness.” The lifecycle is therefore not a sequence of independent problems but a continuous unfolding of the same underlying dynamics. Recognition accelerates centralization; centralization attenuates external engagement; attenuation produces compression; and compression activates restoration.

This recursive progression explains why high-intensity relationships are often experienced as both uniquely meaningful and structurally fragile.

## 6 Mathematical formalization

The Dyadic Universe Model (DUM) can be formalized as a dynamical system in which dyadic centralization, external-world investment, and subjective compression evolve over time. The aim of this formalization is to demonstrate that the lifecycle described in Section 4 arises from a small set of interacting processes rather than from externally imposed stages. We chose to mathematically formalize the Dyadic Universe Model to make its recursive dynamics explicit and testable. Conceptual descriptions alone can illustrate the idea of centralization and compression, but formalization allows us to specify variables, generate falsifiable predictions, and connect with established dynamical systems approaches in relationship science. In simple terms, equations help clarify how changes in one element (such as centralization) feed back into others (such as external engagement and dispositional pressure) over time.

At the same time, we recognize that mathematical formulation introduces limitations. Relationships are complex, subjective, and context-dependent, and no set of equations can fully capture their richness. Formalization risks oversimplifying lived experiences, reducing them to variables that may miss cultural, emotional, or narrative dimensions. We therefore present the mathematical model not as a complete account of relationships, but as a structured starting point for empirical testing. Its value lies in highlighting measurable dynamics and generating hypotheses, while its limits remind us that qualitative and contextual insights remain essential for a full understanding of relational life.

To preserve conceptual clarity, this section presents the core model and its qualitative implications, while detailed derivations, parameter specifications, and extended analyses are provided in the Appendices A–E.

### 6.1 Core dynamical system

The following system represents a formal abstraction of the proposed dynamics, rather than a direct empirical measurement model. The system is represented by three state variables:

- C(t) ε [0,1]: dyadic centralization
- E(t) ε [0,1]: external-world investment
- K(t) ≥ 0: accumulated subjective compression

Subjective compression, introduced conceptually in Sections 3 and 4, is here formalized as an accumulating state variable K(t) capturing the dynamic buildup of perceived restriction under sustained centralization.

The system evolves according to:


$$\begin{array}{lll} \frac{dC}{dt} & = & \alpha \cdot E\cdot \left(1-C\right)-\kappa \cdot C\cdot H\left(K\right)\\ \frac{dE}{dt} & = & \gamma \cdot \left(E_{0}-E\right)-\beta \cdot C\cdot E\\ \frac{dK}{dt} & = & \delta \cdot \psi \cdot C\cdot \left(1-E\right)-\rho \cdot K \end{array}$$

Where H(K) is a threshold function governing the activation of autonomy-restoration processes.

Parameter definitions in Appendix B.

#### 6.1.1 Interpretation of the core system

These equations operationalize the recursive dynamics introduced in Sections 3 and 4:

- *Centralization growth and regulation*. Centralization increases by drawing from available external engagement (E) but is reduced when compression activates autonomy-restoration processes.
- *External-world attenuation*. External investment recovers toward a baseline level but is continuously diminished as centralization increases.
- *Compression dynamics*. Compression accumulates when centralization is high and external engagement is low, with intensity modulated by dispositional pressure (ψ).

Together, the system formalizes the core feedback loop:

C ↑ → E ↓ → compression (K) ↑ → autonomy restoration → C adjusts.

This loop constitutes the generative mechanism of the dyadic-universe regime.

### 6.2 Emergent lifecycle dynamics

The four-phase lifecycle described in Section 4 emerges directly from the system's behavior. From typical initial conditions (low C, high E, negligible K), the system produces a characteristic trajectory:

1. *Rapid increase in centralization*. Corresponding to the recognition and centralization phases, where relational salience intensifies and external engagement declines.
2. *Peak and saturation*. Centralization reaches a maximum as external domains are attenuated, and compression begins to accumulate.
3. *Compression-driven adjustment*. As compression crosses a threshold, autonomy-restoration processes activate, reducing or destabilizing centralization.

A key implication is that the lifecycle is endogenously generated by the model rather than imposed as a descriptive sequence.

#### 6.2.1 Role of dispositional pressure (ψ)

Dispositional pressure (ψ) governs the shape and stability of trajectories:

Higher ψ

- Accelerates compression accumulation
- Triggers earlier activation of autonomy restoration
- Reduces long-term centralization

Lower ψ

- Delays compression
- Allows sustained high-centralization states
- Produces more stable equilibria

This provides a formal account of individual differences in relational trajectories, consistent with propositions P6 and P7.

### 6.3 Dyadic implications

Extending the system to two interacting partners introduces a crucial structural prediction. Differences in dispositional pressure produce asymmetric relational outcomes.

1. In dyads where partners differ in ψ:

The higher-ψ partner

- Accumulates greater compression
- Experiences stronger autonomy-restoration pressure
- The lower-ψ partner

The lower-ψ partner

- Maintains higher levels of centralization
- Experiences less compression at equivalent states

2. This generates a stable asymmetry in which:

- One partner experiences increasing constriction
- The other remains immersed in the dyadic structure

This configuration provides a formal basis for Proposition P13 and represents a key precursor to destabilization in Phase 4. (Detailed dyadic equations and simulations are provided in Appendix D).

### 6.4 Rupture and stochastic extension

The deterministic model converges to equilibrium states; however, real-world relationships may terminate. To capture this, the model incorporates a stochastic extension in which rupture is treated as a probabilistic event.

The central principle is “The model assumes that rupture probability increases as a function of accumulated compression and dispositional pressure.” When compression exceeds a critical threshold, individuals, particularly those high in ψ, are more likely to engage in rupture-prone autonomy-restoration behaviors.

This extension yields two key predictions:

- Initiator asymmetry: In mismatched dyads, the higher-ψ partner is more likely to initiate rupture.
- Timing effects: Higher levels of ψ are associated with shorter time-to-rupture trajectories.

These predictions provide a quantitative foundation for the behaviors described in Section 4 and elaborated in Section 6. (Full stochastic specification and simulation results are provided in Appendix E).

### 6.5 Integration with theoretical propositions

The formal model provides a generative basis for the propositions introduced in Section 3:

- P1–P3

Follow directly from the coupling between centralization (C) and external investment (E)

- P4–P5

Emerge from the transition from expansion to compression as external resources are attenuated

- P6

Derived from the sensitivity of compression dynamics to dispositional pressure (ψ)

- P7–P8

Reflected in the activation and outcomes of autonomy-restoration processes

- P13–P15

Derived from dyadic asymmetry and stochastic rupture dynamics

Importantly, several of these predictions, particularly those concerning asymmetry and rupture initiation, arise only through formalization and are not directly derivable from the verbal model alone.

### 6.6 Summary

The mathematical formalization demonstrates that a minimal set of interacting variables (C, E, K, ψ) is sufficient to generate: (1) the full lifecycle trajectory; (2) individual differences in relational stability; (3) asymmetry in dyadic systems; and (4) probabilistic rupture behavior. The same recursive mechanism accounts for: (1) the emergence of intense relational immersion; (2) the development of subjective compression; and (3) the activation of autonomy-restoration processes. The model therefore functions as a generative system, linking the conceptual structure of Sections 3 and 4 to a set of precise, time-dependent, and empirically testable predictions. (The global sensitivity analysis is presented in Appendix C). These dynamics provide the formal basis for the autonomy-restoration behaviors discussed in Section 6, where threshold-driven adjustments in centralization manifest as observable actions such as reinvestment, distancing, or extra-dyadic involvement.

## 7 Autonomy-restoration behaviors, including infidelity

Autonomy-restoration behaviors are actions through which individuals attempt to regain a sense of independence, novelty, or personal identity after periods of high dyadic centralization. Within the Dyadic Universe Model (DUM), these behaviors are not treated as isolated events, but as predictable responses to the accumulation of subjective compression described in Sections 4 and 5.

### 7.1 Reframing autonomy-restoration

In everyday terms, autonomy-restoration occurs when a person begins to feel that their life has become too narrowly centered on the relationship. This corresponds to Phase 4 (autonomy restoration) in the lifecycle described in Section 4, where accumulated compression activates corrective processes within the dyad. Even if the relationship remains meaningful and loving, the person may experience a growing need to reconnect with other parts of their identity, such as personal goals, friendships, or new experiences.

The model proposes that this process emerges naturally from the same dynamics that create intense closeness. As relationships become increasingly central (high C), engagement with the external world (E) tends to decline. Over time, this reduction limits opportunities for growth and novelty, leading to the buildup of subjective compression (K). For individuals with higher dispositional pressure (ψ), this buildup is experienced more strongly, increasing the likelihood that autonomy-restoration behaviors will occur.

This framing aligns with research showing that unmet needs for autonomy and personal growth can motivate behavioral change even within otherwise satisfying relationships (Ryan and Deci, 2017).

### 7.2 Infidelity as one form of autonomy-restoration

Within this broader category, infidelity is treated as one possible, though not inevitable, form of autonomy-restoration. The model does not suggest that infidelity is justified or desirable. Rather, it is presented as one type of behavior that may arise when individuals attempt to restore novelty, identity diversification, or personal agency. This account does not imply that infidelity is primarily or universally driven by these dynamics but rather identifies one potential pathway among multiple established explanations.

This perspective helps explain a pattern frequently observed in empirical research: that infidelity sometimes occurs in relationships that individuals still describe as meaningful or emotionally significant (Fincham and May, 2017). Traditional explanations often attribute infidelity primarily to dissatisfaction, poor relationship quality, or opportunity. While these factors are important, they do not fully account for cases where individuals report both deep attachment and a simultaneous desire for external engagement.

From the model's perspective, these cases reflect a structural tension: the relationship has become highly central, but the individual's dispositional need for expansion remains unresolved. Within the model, such behaviors emerge when accumulated compression (K) exceeds a threshold sufficient to activate autonomy-restoration processes, as formalized in Section 5.

### 7.3 Two pathways of autonomy-restoration

The DUM distinguishes between constructive and destabilizing forms of autonomy-restoration.

**(a) Constructive restoration**

These behaviors help rebalance the individual's life without undermining the relationship:

- Reinvesting in friendships or professional roles
- Pursuing hobbies or personal interests
- Establishing healthier boundaries within the relationship
- Engaging in shared but outward-facing activities

Such behaviors increase external-world investment (E) and reduce compression without necessarily reducing relational closeness. Research on self-expansion supports the idea that introducing new activities into a relationship can improve satisfaction and maintain vitality (Aron et al., 2000; Muise et al., 2019).

**(b) Destabilizing restoration**

Other forms of autonomy-restoration may disrupt or undermine the relationship:

- Emotional distancing
- External validation seeking
- Secretive behaviors or deception
- Extra-dyadic involvement (including infidelity)

These behaviors often occur when constructive restoration is unavailable, discouraged, or delayed. In such cases, the individual seeks relief from compression through less integrated or more reactive means.

### 7.4 Dyadic asymmetry in restoration behaviors

The dyadic extension of the model (Section 5.3; Appendix D) and the stochastic extension (Section 5.4; Appendix E) together predict that autonomy-restoration does not occur equally across partners.

In relationships where partners differ in dispositional pressure (ψ):

- The higher-ψ partner

° Experiences stronger compression
° Is more likely to initiate autonomy-restoration

- The **lower-ψ** **partner**

° Maintains higher tolerance for centralization
° Is less likely to initiate change

This asymmetry provides a structural explanation for imbalanced patterns such as one partner pursuing change while the other resists it. It also aligns with research on demand–withdraw interaction patterns in relationships (Christensen and Heavey, 1990; Schrodt et al., 2014), though the DUM situates this pattern within longer-term identity dynamics rather than momentary interaction cycles.

### 7.5 Practical and research implications

This framework leads to several practical implications:

1. **Intervention focus**

Relationship problems in high-intensity dyads may not stem from lack of love or commitment, but from insufficient external engagement. Interventions that only focus on improving intimacy may therefore miss the underlying issue.

2. **Prevention of destabilization**

Allowing and normalizing external-world reinvestment early in the relationship may reduce the buildup of compression and lower the likelihood of destabilizing behaviors.

3. **Multiple pathways model**

Autonomy-restoration behaviors should be understood as having at least two distinct causes:

° Dissatisfaction-driven
° Centralization-driven (as proposed here)

These pathways may lead to different outcomes and require different interventions.

### 7.6 Summary

In the Dyadic Universe Model, autonomy-restoration behaviors, whether constructive or destabilizing, are not random or purely situational. They emerge from the same recursive system that produces high relational intensity.

Simply put:

The closer the relationship becomes to being “everything,” the more likely it is that individuals will eventually seek something beyond it. This reframing helps explain why deeply meaningful relationships can also become unstable, and why behaviors such as distancing or infidelity can arise even in relationships that remain emotionally significant. These behaviors therefore represent the observable manifestation of the Phase 4 transition in the lifecycle, driven by accumulated compression and moderated by dispositional pressure (ψ).

## 8 Research agenda

To empirically evaluate the Dyadic Universe Model (DUM), we propose a coordinated program of research that examines how relationships evolve when they become highly central within an individual's life system. Across studies, the focus is on the interaction of four core elements: dyadic centralization (C), external-world investment (E), subjective compression (K), and dispositional pressure (ψ).

Rather than treating relationships as static, this research program examines how they unfold across the four lifecycle phases described in Section 4: recognition, centralization, saturation, and autonomy restoration. Each study isolates a different level of analysis, from measurement to long-term development to observable behaviors, while remaining anchored in the same underlying recursive dynamics.

### 8.1 Study 1. Construct validation

The first study establishes reliable measurement of the model's core components. Specifically, it develops and validates scales capturing:

- **Dyadic centralization (C)** – the degree to which the relationship becomes the primary source of identity and engagement.
- **External-world investment (E)** – continued participation in social, occupational, and identity-relevant domains outside the relationship.
- **Dispositional pressure (ψ)** – the individual's drive for novelty, growth, and diversification.
- Derived constructs such as relational salience and partner-mediated identity restructuring.

To ensure discriminant validity, these measures will be tested against established constructs, including attachment dimensions, self-expansion, differentiation of self, personality traits, and relationship satisfaction.

The purpose of this study is foundational: before testing the model's predictions, the constructs themselves must be shown to capture processes that are empirically distinct yet theoretically coherent.

### 8.2 Study 2. Dyadic longitudinal panel (lifecycle trajectories)

The second study examines how relationships evolve over time as they move through the lifecycle phases of the model. Newly formed couples are followed longitudinally, with repeated measurement of C, E, K, and ψ for both partners. This design directly tests the central lifecycle predictions:

- Whether relationships exhibit rapid early increases in centralization (recognition and centralization phases).
- Whether increases in centralization correspond to declines in external investment (E ↓).
- Whether sustained centralization leads to the emergence of subjective compression (K ↑) (saturation phase).
- Whether autonomy-seeking or corrective behaviors appear after peak closeness (autonomy-restoration phase).

The dyadic structure allows examination of asymmetry, particularly whether differences in dispositional pressure (ψ) predict divergent trajectories between partners.

This study provides the primary empirical test of whether the lifecycle described in Section 4 emerges in real-world relationships.

### 8.3 Study 3. Experience-sampling study (micro-level dynamics)

The third study captures how the model operates in everyday life using experience-sampling methods. Participants report moment-to-moment or daily experiences over a defined period, including:

- Fluctuations in relational focus and centralization (C)
- Perceived restriction or compression (K)
- Desire for independence, novelty, or external engagement
- Autonomy-restoration behaviors (e.g., seeking space, engaging outside the relationship)

This study tests whether increases in compression predict subsequent autonomy-restoration tendencies, particularly among individuals high in **ψ**. Importantly, this design allows direct observation of the recursive process: **C** **↑** **→** **E** **↓** **→** **K** **↑** **→** **autonomy-restoration** as it unfolds in real time.

### 8.4 Study 4. Dyadic experimental study (intervention and causal tests)

The fourth study tests whether the model's dynamics can be altered through targeted intervention. Couples in high-centralization states are randomly assigned to one of three conditions:

- **Intimacy-focused intervention** (standard relational approach)
- **External-engagement intervention** (encouraging reinvestment in external domains, increasing E)
- **Combined intervention**

Outcomes include changes in centralization (C), compression (K), and overall relational stability.

This study evaluates a central implication of the model: “Relational strain in high-intensity dyads may arise not from insufficient intimacy, but from insufficient external-world engagement. If supported, the findings would demonstrate that restoring balance between C and E can reduce compression and improve stability.”

### 8.5 Study 5. Survival and outcome analysis (destabilization and rupture)

The fifth study examines long-term outcomes in high-intensity relationships, focusing on the transition from autonomy-restoration to potential destabilization or rupture.

Key variables include:

- Accumulated compression (K) over time
- Dispositional pressure (ψ)
- Behavioral manifestations of autonomy-restoration (from constructive reinvestment to destabilizing actions such as distancing or extra-dyadic involvement)

The study tests whether:

- Individuals higher in **ψ** are more likely to initiate autonomy-restoration behaviors
- The interaction of compression and ψ predicts the likelihood of destabilizing outcomes
- These processes occur even in relationships that remain emotionally meaningful

This provides a direct empirical test of the model's central claim:

The same processes that produce deep relational immersion can generate conditions for instability.

#### 8.5.1 Integration across studies

Taken together, these studies examine the Dyadic Universe Model across multiple levels of analysis:

- **Measurement level:** establishing reliable constructs for C, E, ψ, and K.
- **Developmental level:** testing whether relationships follow the predicted lifecycle trajectory.
- **Experiential level:** capturing the recursive dynamics of compression and restoration in daily life.
- **Intervention level:** assessing whether modifying external engagement alters outcomes.
- **Outcome level:** explaining variability in autonomy-restoration behaviors and relationship stability.

Across all studies, the central theoretical prediction remains consistent:

As dyadic centralization increases (C ↑) and external-world investment declines (E ↓), subjective compression (K) accumulates, particularly among individuals high in dispositional pressure (ψ), thereby increasing the likelihood of autonomy-restoration processes.

#### 8.5.2 Methodological considerations

Given the novelty of the Dyadic Universe Model, empirical testing requires rigorous design. We recommend that studies:

- Employ dyadic data, capturing both partners' trajectories
- Incorporate longitudinal and experience-sampling methods to observe temporal dynamics
- Utilize pre-registration and open data practices to ensure transparency and replicability

These methodological commitments are essential to evaluate whether the DUM provides explanatory power beyond existing frameworks in relationship science. Given the model's novelty, initial empirical tests should be interpreted cautiously, with emphasis on replication and convergence across methods.

## 9 Discussion

### 9.1 Clinical implications: balancing closeness and independence

The Dyadic Universe Model (DUM) explains why relationships can feel both deeply meaningful and emotionally overwhelming. It shows that difficulties are not only about poor communication or lack of affection, but often stem from structural imbalances in dyadic centralization (C), external-world engagement (E), and compression (K). When couples become too immersed in each other (high C, low E), they may lose touch with outside identities, which creates distress. Clinical studies suggest a related pattern: being “too immersed” often sustains symptoms, while learning to step back reduces emotional strain (Barbosa et al., 2017).

Therapy research on pathological dependence and fear of intimacy highlights how people may cling to closeness or avoid it, both reflecting the DUM's cycle of centralization and autonomy restoration (Papa et al., 2026; Incekara and Ulaş, 2026). Reviews of codependency also show how identity and functioning can break down when the relationship becomes the sole focus (Molina et al., 2026).

The model predicts (P9) that effective intervention depends on restoring balance between closeness and independence, rather than simply increasing intimacy. Clinical assessment should therefore evaluate:

- The degree of centralization (C),
- The extent of external engagement (E),
- Indicators of compression (K), and
- Dispositional differences (ψ).

Importantly, dispositional mismatches (P13–P15) mean that one partner may accumulate compression faster, increasing rupture risk. This suggests therapy should not treat partners symmetrically but identify where instability is structurally concentrated.

### 9.2 Digital-era amplification: technology as accelerator

Modern digital technologies intensify these dynamics by accelerating centralization and reducing external engagement. Constant texting, video calls, and social media increase the growth rate of centralization (α) and the attenuation of external domains (β). As shown in sensitivity analyses, α strongly influences peak centralization, while ψ shapes long-term compression. This leads to prediction (P10): technologies that heighten α advance the onset of compression and autonomy-restoration processes.

Empirical studies support this. Digital communication can make relationships easier to maintain but also risks privacy loss, online abuse, or reduced satisfaction if overused (Daspe et al., 2026; Munshi et al., 2024). At the same time, new tools like virtual reality show promise in strengthening intimacy, with VR-based touch experiments increasing trust (Ha and Sharabi, 2026).

Thus, the recursive mechanism of the DUM remains intact [C ↑ → E ↓ → K ↑ → corrective adjustment] but digital environments compress the timeline, producing faster transitions across phases. The model helps explain both the risks and opportunities of technology in shaping relational intensity.

### 9.3 Cultural and gender variability: contextual modulation

Although the DUM is structurally gender-neutral, cultural and social contexts shape how its parameters unfold. Two are especially sensitive:

- γ (external recovery rate), reflecting the availability and legitimacy of external roles, and
- The normative moderator in P8, which governs the perceived legitimacy of autonomy-restoration behaviors.

In collectivist cultures, love often emphasizes duty and sacrifice, making centralization feel natural or expected (Walker and Pervez, 2026). Where external engagement is constrained (low γ), compression accumulates more quickly. Similarly, in contexts where autonomy-seeking is discouraged, restoration may be delayed or expressed indirectly. Scholars note that relationship science often overlooks these cultural forces (Kawas and Ogolsky, 2023).

Gender norms and heteronormative expectations also influence how closeness and autonomy are negotiated, affecting when and how compression or autonomy restoration occurs (Few-Demo and Allen, 2023). Accordingly, the model predicts (P11–P12) that cultural and gender factors alter the timing and expression of autonomy-restoration behaviors, while leaving the recursive mechanism intact. Future research should compare these variations to assess how universal or culture-specific the DUM truly is.

### 9.4 Theoretical integration

A central contribution of the Dyadic Universe Model (DUM) is the integration of relational phenomena within a single dynamic system defined by C (centralization), E (external engagement), ψ (dispositional pressure), and K (compression). Constructs often treated independently—such as identity fusion, relational intensity, stagnation, and destabilization—are here understood as sequential manifestations of the same recursive process.

In this framework:

- Rapid increases in C generate intense relational meaning,
- Declines in E limit access to external sources of novelty and growth,
- Accumulation of K produces subjective compression, and
- Autonomy-restoration behaviors emerge as corrective responses.

This integration supports P5 (inverted-U effect of centralization) and P7–P8 (autonomy-restoration dynamics), showing that intensity and instability are not opposing outcomes but co-products of a single system.

At the same time, the DUM connects with established theories of human relationships. Attachment theory explains how early bonding patterns shape later tendencies toward closeness or autonomy (Bowlby, 1988). Sternberg's triangular theory of love highlights the interplay of intimacy, passion, and commitment, which parallels the DUM's phases of centralization and autonomy restoration (Sternberg, 1986). Systems theory further reminds us that relationships are embedded in ecological contexts, where external roles and identities influence dyadic balance (Bronfenbrenner, 1979). By integrating these frameworks, the DUM's cycle of closeness and independence is not speculative but an extension of well-documented relational dynamics.

### 9.5 Methodological implications

The DUM emphasizes that relationship outcomes cannot be predicted solely from stable individual traits. Instead, it highlights the interaction between dispositional pressure (ψ) and dynamic state variables (C, E, K). This perspective aligns with evidence that relationship trajectories exhibit substantial heterogeneity, shaped by both intra-dyadic processes and contextual factors.

The model does not claim deterministic prediction but specifies conditional mechanisms through which relational patterns emerge. Its contribution lies in providing a structured framework for generating empirically testable hypotheses about how these processes unfold over time.

Testing the DUM requires methodological tools capable of capturing both partners' experiences and the recursive nature of dyadic processes. Dyadic data analysis techniques, such as the Actor–Partner Interdependence Model (APIM), allow researchers to examine how one partner's centralization or compression influences the other (Kenny et al., 2006). Longitudinal designs have been used to trace relationship trajectories over time, showing how closeness and autonomy unfold across months or years. Mixed-methods approaches, combining surveys with qualitative interviews, are also recommended for capturing both structural variables and lived experiences (Creswell and Plano Clark, 2017). These methodological precedents demonstrate that the DUM can be empirically tested using established tools, ensuring that its claims are research-ready.

### 9.6 Future direction and limitations

Several limitations should be acknowledged. First, the propositions derived from the model (P1–P15) remain to be empirically tested. Second, the formal system relies on theoretically motivated parameter values rather than empirical estimation. Third, predictions are bounded by specific conditions: dyads in which both partners are low in dispositional pressure (ψ) may sustain stable high-centralization equilibria without significant compression. The current formulation is restricted to dyadic relationships and does not extend to more complex relational structures. Additionally, while the stochastic extension models rupture as a discrete event, real-world autonomy-restoration processes may unfold along a continuum, including reinvestment and boundary renegotiation prior to destabilization.

These limitations can be reinforced by engaging with current reviews and meta-analyses. Research on relationship trajectories emphasizes the importance of comparing models across diverse populations and cultural contexts (Ogolsky et al., 2013). Meta-analyses on digital intimacy point to gaps in understanding how technology reshapes closeness and autonomy (Ruppel and Burke, 2015). Scholars also caution that models must account for intersectional factors such as socioeconomic status, gender norms, and cultural scripts (Few-Demo and Allen, 2023). These insights suggest that the DUM's phases: recognition, centralization, saturation, and autonomy restoration, should be tested across varied contexts to determine whether they are universal or culture-specific. Recognizing these limitations ensures that the model remains open to refinement and avoids overstating its scope.

## 10 Conclusion

The Dyadic Universe Model (DUM) proposes that high-intensity romantic relationships can be understood as a distinct relational regime governed by a minimal recursive system defined by dyadic centralization (C), external-world investment (E), dispositional pressure (ψ**)**, and subjective compression (K). Within this system, a single generative principle is proposed to generate a wide range of relational phenomena: “Increasing centralization reduces engagement with external domains, generating compression that ultimately acts back upon centralization.” From this principle, the model derives a characteristic four-phase lifecycle: recognition, centralization, saturation, and autonomy restoration, as well as a formal dynamical system in which these phases emerge endogenously. It further specifies a set of falsifiable propositions (P1–P15) that address nonlinear escalation of intensity, the transition from expansion to compression, asymmetry in dyadic systems, and probabilistic rupture dynamics.

The central theoretical contribution of the model is the unification of relational intensity and structural fragility within a single explanatory framework. Rather than treating closeness and instability as separate phenomena, the DUM demonstrates that they arise from the same underlying recursive dynamics. In this sense, the processes that produce exceptional relational immersion also generate the conditions that limit its sustainability.

Importantly, the model does not claim inevitability. Not all relationships enter the dyadic-universe regime, and not all that do become unstable. Instead, it specifies the conditions under which high levels of centralization (C ↑), reduced external engagement (E ↓), and elevated dispositional pressure (ψ ↑) lead to the accumulation of compression (K), thereby increasing the likelihood of autonomy-restoration processes.

The Dyadic Universe Model thus reframes a familiar paradox in close relationships: “Deep relational immersion is not inherently unstable; however, when it becomes structurally exclusive, it generates the forces that constrain its long-term viability.”

The validity of this account remains an empirical question. The research program outlined in Section 8 provides a systematic approach to testing the model's predictions across measurement, longitudinal, experiential, and intervention-based designs. The DUM is therefore offered as a parsimonious, formally grounded, and empirically testable framework for advancing theoretical and empirical work in the science of romantic relationships.

## Data Availability

The original contributions presented in the study are included in the article/Supplementary material, further inquiries can be directed to the corresponding author.
