# Supplementary material for: The Dyadic Universe Model: a process theory of centralization, compression, and autonomy in romantic relationships
Source: Front Psychol. 2026 Oct 19;17:1935191. doi: 10.3389/fpsyg.2026.1935191 (PMC13724465; doi:10.3389/fpsyg.2026.1935191)
Supplement: Supplementary file 1 [file Supplementary_file_1.docx]

**Appendix A: Analytical Derivations**

***A.1 Equilibria and stability***

Setting (5.1)–(5.3) to zero yields the interior equilibrium $\left( C^{*} , E^{*} , K^{*} \right)$.

From (5.2):

E* = γ·E₀ / (γ + β·C*)	(A.1)

From (5.3):

K* = (δ·ψ / ρ) · C* · (1 − E*)	(A.2)

Substitution into (5.1) yields a reduced equation in $C^{*}$, whose solution exists and is unique in (0,1) under admissible parameter values.

***A.2 Jacobian and Eigenvalues***

The Jacobian evaluated at equilibrium $J(C^{*},E^{*},K^{*})$defines local system behavior.

For the default parameter set (ψ = 0.6), eigenvalues are:

λ₁ ≈ −0.19

λ₂,₃ ≈ −0.19 ± 0.20i	(A.3)

***A.3 Stability Classification***

The system exhibits:

- **Stable focus (damped oscillation)** when eigenvalues are complex with negative real part
- **Overdamped convergence** when eigenvalues are real and negative

For higher ψ (e.g., ψ = 2.2, 5.0), all eigenvalues become real and negative.

The transition between regimes occurs near: ψ ≈ 1.0	(A.4)

These stability regimes correspond to the lifecycle dynamics described in Section 4. In particular, oscillatory convergence reflects repeated adjustment cycles in autonomy-restoration, while overdamped convergence corresponds to more direct transitions toward stable or destabilized states depending on dispositional pressure (ψ).

***A.4 Feedback Specification***

To make the recursive nature of the Dyadic Universe Model explicit, autonomy‑restoration behaviors are modeled as recursive inputs that alter subsequent states of centralization (C) and external investment (E). Formally, if autonomy‑restoration occurs at time t, then centralization at time t+1 is adjusted by ΔC, and external investment at time t+1 is adjusted by ΔE:

$$>C_{t+1}=C_{t}-\Delta C ; E_{t+1}=E_{t}+\Delta E>$$

where ΔC represents the reduction in dyadic intensity due to corrective withdrawal, and ΔE represents the re‑opening of external opportunities. Conversely, failed autonomy‑restoration attempts increase dispositional pressure (ψ), which feeds back into compression (K) and can reinforce centralization rather than reduce it:

$$>C_{t+1}=C_{t}+f\left( \psi\right) ; E_{t+1}=E_{t}-g\left( \psi\right)>$$

This recursive specification ensures that autonomy‑restoration is not treated as a terminal outcome but as a dynamic input that reshapes subsequent trajectories of C and E. The oscillatory or convergent patterns observed in the stability regimes (Appendix A) reflect these recursive adjustments, distinguishing the DUM from a unidirectional mediation chain.

**Appendix B: Parameters**

***B.1 Table 2. Model parameters and their psychological interpretation.***

| **Parameter** | **Symbol** | **Interpretation** | **Range** |
| --- | --- | --- | --- |
| Restructuring responsiveness | α | Rate at which available external resources drive centralization growth | 0.05–0.6 |
| Centralization-driven attenuation | β | Rate at which centralization absorbs external investment | 0.1–0.5 |
| External recovery | γ | Rate at which external investment recovers toward baseline | 0.02–0.20 |
| Baseline external investment | E₀ | Pre-relationship engagement with external domains | 0.7–1.0 |
| Compression generation | δ | Rate at which attenuated centralization generates compression | 0.1–0.4 |
| Compression dissipation | ρ | Rate of adaptation to accumulated compression | 0.03–0.15 |
| Autonomy-restoration intensity | κ | Maximum rate of compression-driven centralization suppression | 0.4–1.5 |
| Compression threshold | Kₕ | Half-saturation point of autonomy-restoration activation | 0.4–1.2 |
| Threshold steepness | n | Hill exponent governing sharpness of restoration onset | 2–8 |
| Composite dispositional pressure | ψ = φ + ε | Fluidity plus existential expansion need; per-individual constant | 0–8 |

Variations in these parameters correspond to differences in lifecycle trajectories described in Section 4. For example, higher α accelerates the recognition and centralization phases, while higher β and lower γ increase the likelihood of entering saturation. Dispositional pressure (ψ) plays a central role in determining the timing and intensity of autonomy-restoration.

**Appendix C: Sensitivity Analysis**

***C.1 5.2 Method - Global sensitivity analysis***

Use of Sobol indices (S₁ and Sₜ)

Parameter space: (α, β, γ, δ, ρ, κ, Kₕ, ψ)

Sampling method:

- Saltelli extension
- $N_{base}=1024$
- Total runs: 10,240

Simulation setup:

- Time horizon: 180 months
- Outputs measured:
  - Peak centralization
  - Long-run centralization
  - Long-run compression

***C.2 Sensitivity Results (Sobol Indices)***

*Figure 2. Global variance-based sensitivity indices for the single-individual deterministic model.*

Figure 2 reports first-order (S₁) and total-order (Sₜ) Sobol indices for peak centralization, long-run centralization, and long-run compression. Blue bars represent first-order Sobol indices (S₁), and orange bars represent total-order indices (Sₜ) for peak centralization, long-run centralization, and long-run compression.

***C.3 Parameter Sweeps***

*Figure 3. One-at-a-time parameter sweeps.*

Figure 3 displays one-at-a-time parameter sweeps showing the response of peak centralization, long-run centralization, and long-run compression to variation in each parameter. Green curves represent long-run centralization, and red curves (right axis) represent long-run compression. The vertical dotted line indicates the default parameter value.

These results reinforce the theoretical claims developed in Sections 3–6. In particular, the dominant role of ψ in long-run compression aligns with Proposition P6 and the autonomy-restoration dynamics in Section 6, while the influence of α on peak centralization corresponds to the rapid escalation described in Phase 1–2 of the lifecycle (Section 4).

**Appendix D: Dyadic System (Full Formulation)**

***D.1 Coupled Dyadic System Equations***

dCᵢ/dt = αᵢ·Eᵢ·(1−Cᵢ) + μ·Cⱼ·(1−Cᵢ) − κᵢ·Cᵢ·H(Kᵢ)	(D.1)

dEᵢ/dt = γᵢ·(E₀ᵢ − Eᵢ) − βᵢ·Cᵢ·Eᵢ	(D.2)

dKᵢ/dt = δ·ψᵢ·Cᵢ·(1−Eᵢ) − ρ·Kᵢ	(D.3)

These equations represent the coupled two-partner extension of the DUM system, with i, j ∈ {1,2}, i ≠ j.

***D.2 Dyadic Configurations (Inputs Only)***

Three configurations were simulated:

1. Symmetric matched: ψ₁ = ψ₂ = 2.0

2. Asymmetric mismatch: ψ₁ = 0.5, ψ₂ = 4.5

3. Symmetric high: ψ₁ = ψ₂ = 4.5

***D.3 Simulation Results***

*Figure 4. Coupled dyadic system trajectories under three parameter configurations.*

Solid and dashed lines represent the two partners. Panels display centralization, external investment, compression, and cross-partner phase trajectories.

Raw equilibrium values:

C₁* ≈ 0.45, C₂* ≈ 0.23

K₁* ≈ 0.59, K₂* ≈ 1.96

These asymmetric configurations provide the structural basis for the behavioral patterns described in Section 6, where the higher-ψ partner is more likely to initiate autonomy-restoration behaviors, including distancing or destabilizing actions. The model thus links dispositional asymmetry to observable relational dynamics.

**Appendix E. Stochastic Extension of the Dyadic Model**

***E.1 Stochastic Equations***

$$\begin{matrix} & dC_{i}=[\text{drift}_{C_{i}}]\text{ }dt+\sigma_{C}\cdot C_{i}(1-C_{i})\text{ }dW_{i}^{C} & & \text{(E.1)} \end{matrix}$$

$$\begin{matrix} & dE_{i}=[\text{drift}_{E_{i}}]\text{ }dt+\sigma_{E}\cdot E_{i}(1-E_{i})\text{ }dW_{i}^{E} & & \text{(E.2)} \end{matrix}$$

$$\begin{matrix} & dK_{i}=[\text{drift}_{K_{i}}]\text{ }dt+\sigma_{K}\cdot\sqrt{K_{i}+0.01}\text{ }dW_{i}^{K} & & \text{(E.3)} \end{matrix}$$

The drift terms correspond to the deterministic dyadic system.
$W_{i}^{C}$, $W_{i}^{E}$, and $W_{i}^{K}$are independent Wiener processes.

***E.2 Rupture Hazard Function***

$$\begin{matrix} & h_{i}(t)=\lambda_{0}+\lambda_{1}\cdot\max\left( K_{i}(t)-K_{\text{act}},0 \right)\cdot\psi_{i} & & \text{(E.4)} \end{matrix}$$

The first rupture event in either partner terminates the system.

***E.3 Simulation Setup***

Euler–Maruyama discretization

Time step: $dt=0.05$

Time horizon: 180 months

Monte Carlo replications per configuration.

***E.4 Simulation Results***

*Figure 5. Stochastic dyadic model results. (a₁–a₃) Sample trajectories of partner-1 centralization for symmetric dyads with ψ = 1.0, 3.0, 5.0. Thin lines represent individual stochastic realizations; the thick line represents the mean trajectory. The dashed line indicates a reference level of centralization. (b) Symmetric rupture probability by 15 years as a function of shared ψ. (c) Asymmetric rupture probability with ψ₁ = 0.5 and ψ₂ varied. Black line represents total rupture probability; blue squares and red triangles indicate rupture initiation by partner 1 and partner 2, respectively. (d) Median time-to-rupture for symmetric dyads across values of ψ.*

Thin lines represent individual stochastic realizations; thick lines represent mean trajectories. Panels display centralization trajectories, rupture probability, initiator frequency, and time-to-rupture.

***E.5 Output Measures***

- Rupture occurrence
- Initiating partner
- Time-to-rupture

Although modeled here as discrete rupture events, these stochastic transitions correspond to the broader class of autonomy-restoration behaviors described in Section 6. In practice, the same compression-driven dynamics may manifest in less extreme forms, including reinvestment or boundary renegotiation, prior to rupture.
